# Supplementary material for: Gaussian Mixture Model-Based Focused Refinement for Enhanced Flexible Structure Determination in CryoEM and CryoET
Source: ArXiv. 2026 May 28:arXiv:2605.30518v1. Preprint. [Version 1] (PMC13232415)
Supplement: Supplement 1 — Figure S1. Workflow of GMM-based focused refinement for single particle CryoEM (left) and CryoET (right). Figure S2. Performance of the two optimization methods on a simulated dataset. Different types of dynamics are simulated for the transmembrane domain (cyan mask). The results are evaluated by the FSC between focus-refined structure and the ground truth under the cyan mask. Figure S3. Data processing workflow for the mitochondrial ATP synthase CryoET dataset. [file NIHPP2605.30518v1-supplement-1.pdf]

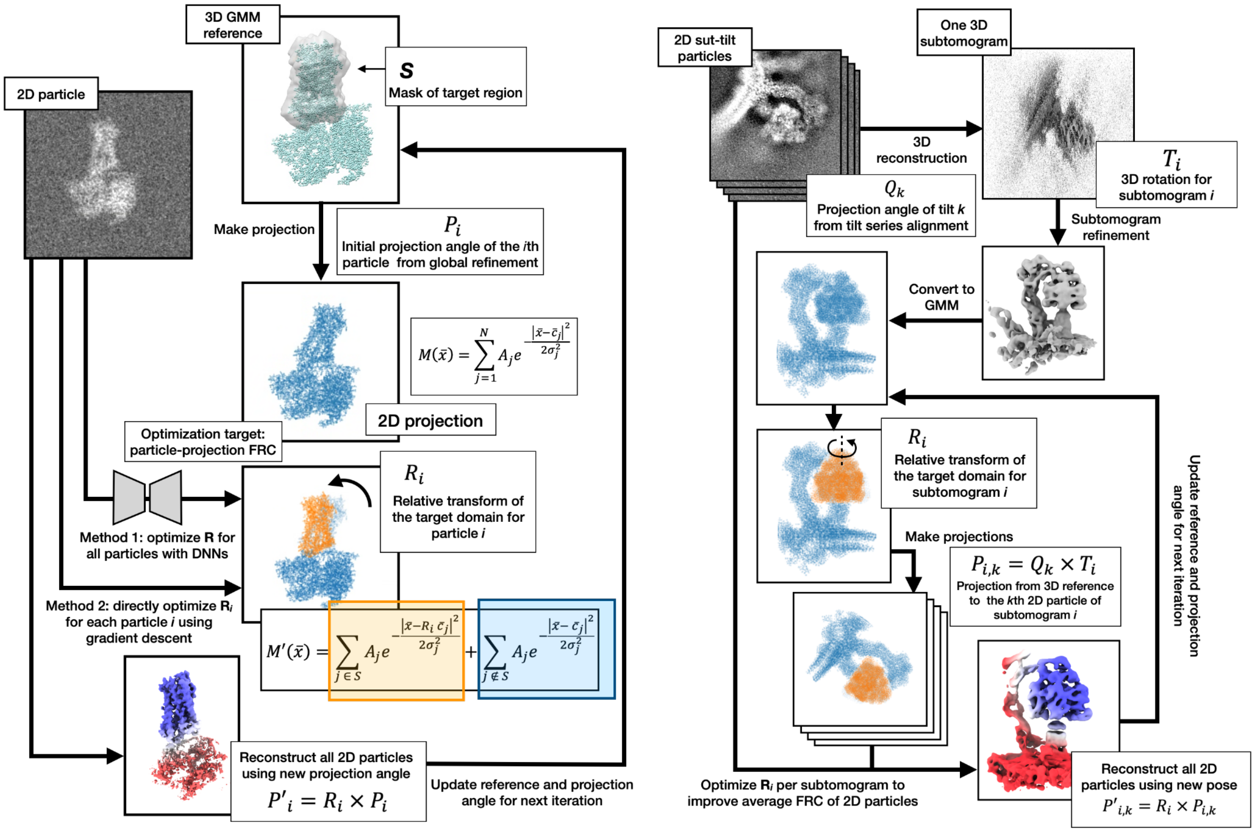

Figure S1. Workflow of GMM-based focused refinement for single particle CryoEM (left) and CryoET (right).

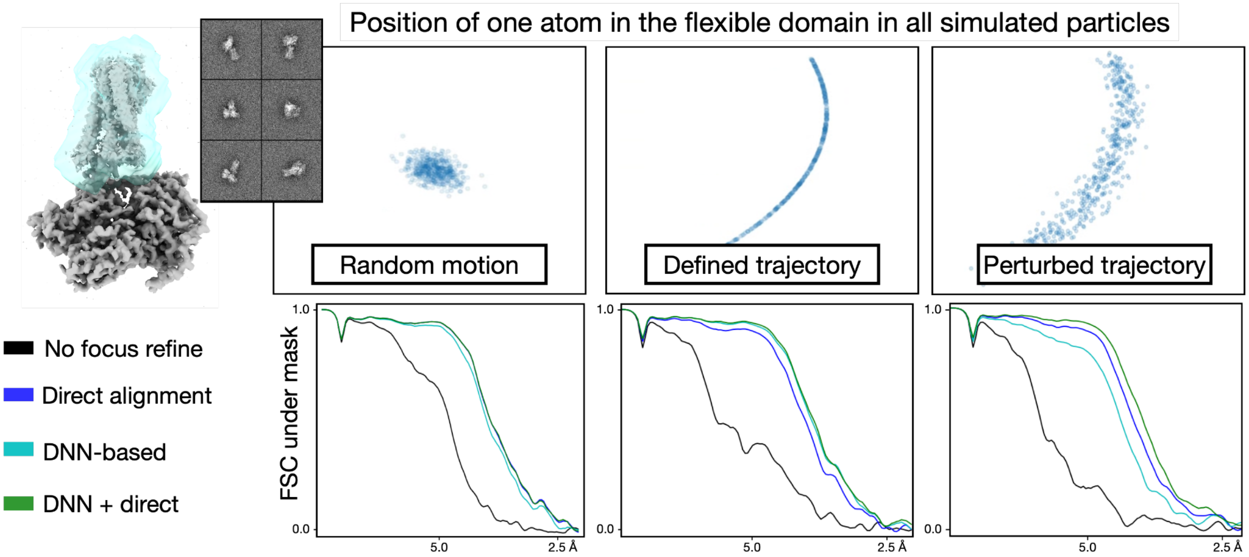

Figure S2. Performance of the two optimization methods on a simulated dataset. Different types of dynamics are simulated for the transmembrane domain (cyan mask). The results are evaluated by the FSC between focus-refined structure and the ground truth under the cyan mask.

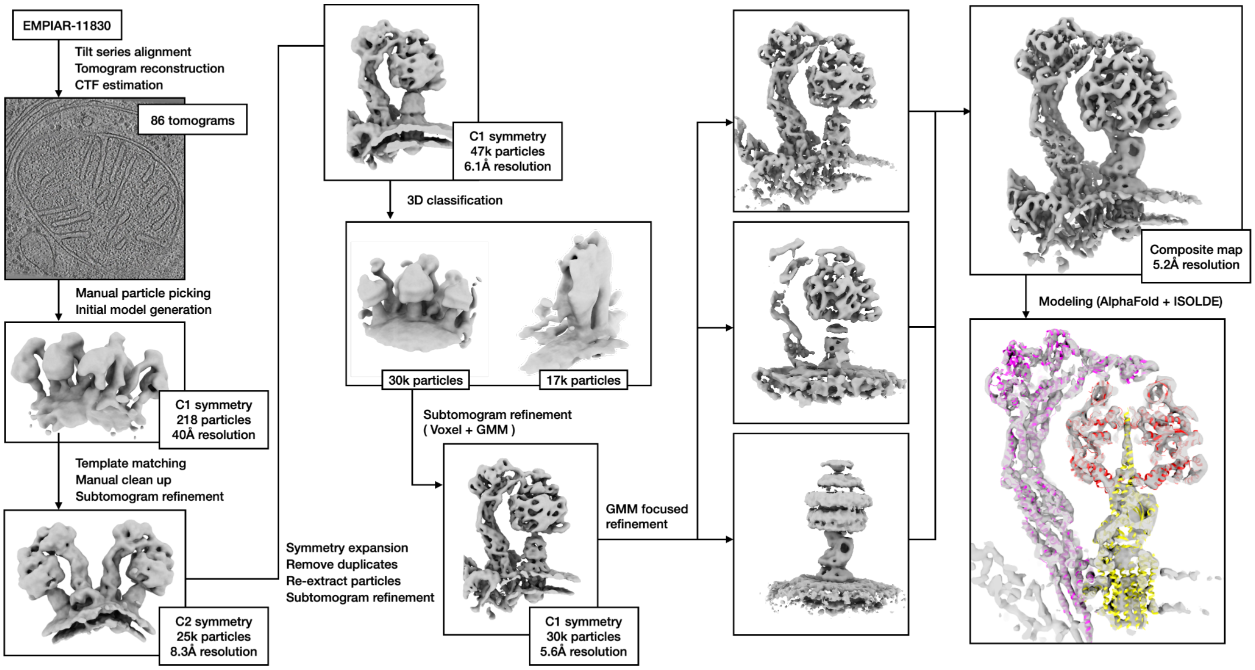

Figure S3. Data processing workflow for the mitochondrial ATP synthase CryoET dataset.
